# Longitudinal associations of intra‐individual cognitive variability with dementia, cognitive change and PET amyloid‐ß in adults with Down syndrome: data from the Alzheimer's Biomarker Consortium‐ Down Syndrome (ABC‐DS)

**DOI:** 10.1002/alz70856_107396

**Published:** 2026-01-09

**Authors:** Luciana Mascarenhas Fonseca, Naomi Sage Chaytor, Michal S Beeri, Eardi Lila, Ali Shojaie, Jasmeer P. Chhatwal, Orestes Vicente Forlenza, Elizabeth Head, Mark Mapstone, Ann D Cohen, Bradley T Christian, Benjamin L Handen, Beau Ances, Thomas J Grabowski, Shahid Zaman

**Affiliations:** ^1^ Washington State University, Spokane, WA, USA; ^2^ Elson S. Floyd College of Medicine, Washington State University, Spokane, WA, USA; ^3^ Herbert and Jacqueline Krieger Klein Alzheimer's Research Center at Rutgers Brain Health Institute, New Brunswick, NJ, USA; ^4^ University of Washington, Seattle, WA, USA; ^5^ Brigham and Women's Hospital, Boston, MA, USA; ^6^ Massachusetts General Hospital, Harvard Medical School, Boston, MA, USA; ^7^ University of Sao Paulo, Sao Paulo, SP, Brazil; ^8^ University of California, Irvine, Irvine, CA, USA; ^9^ Department of Psychiatry, University of Pittsburgh, Pittsburgh, PA, USA; ^10^ Wisconsin Alzheimer's Disease Research Center, University of Wisconsin School of Medicine and Public Health, Madison, WI, USA; ^11^ University of Pittsburgh, Pittsburgh, PA, USA; ^12^ Washington University in St. Louis School of Medicine, St. Louis, MO, USA; ^13^ University of Cambridge, Cambridge, United Kingdom

## Abstract

**Background:**

Because of the high incidence of Alzheimer's disease (AD) amyloid‐b pathology in individuals with Down syndrome (DS), they are considered an ideal target population for anti‐amyloid therapy trials. However, not all individuals with DS develop dementia. Intra‐individual cognitive variability (IICV) is proposed to be a sensitive marker for pre‐clinical changes in AD, but has not yet been tested in individuals with DS. Using the ABC‐DS (Alzheimer's Biomarker Consortium‐DS) data, we characterize the relationships between baseline IICV with dementia status, cognitive decline and brain amyloid‐b (using positron emission tomography (PET)) in adults with DS.

**Methods:**

Data from the ABC‐DS study, includes 460 adults with DS ranging from 25 to 81 years of age (mean age 43.27, 45.7% female) at baseline and mean follow up of 40 months (standard deviation of 7.8 months). All participants had complete neuropsychological evaluations at baseline and follow up, and consensus dementia diagnosis and brain PET amyloid‐b measures in centiloids. IICV measures for memory, executive function and combined memory and executive functions were calculated. Regression models were used to examine the associations between baseline IICV scores with dementia at follow up, clinical presentation worsening from baseline to follow up, more than one standard deviation below the mean change in general cognition considering the level of intellectual disability, as well as PET amyloid‐b at baseline and follow up, adjusting for age, sex, intellectual disability, site, presence of APOEe4 and time latency between baseline and follow up. We adjusted for multiple comparisons using Bonferroni correction.

**Results:**

IICV for memory and executive functions was associated with all outcomes tested (odds ratio ranged from 4.4 to 17.2, *p* <0.05), except for PET amyloid‐b at follow up. The associations of baseline IICV and dementia at follow up persisted with the additional adjustment of cognitive performance on the tests included in the IICV measure.

**Conclusion:**

IICV appears be an early indicator of dementia in individuals with DS. Future studies are needed to understand whether IICV can contribute to clinical trials assessing potential candidates for secondary prevention and what are the underlying mechanisms related to within‐person cognitive variability and the development of AD neuropathology in this population.

